# 循环肿瘤DNA状态与非小细胞肺癌术后中枢神经系统复发风险与总生存的研究进展

**DOI:** 10.3779/j.issn.1009-3419.2026.101.15

**Published:** 2026-07-20

**Authors:** Dandan GAO, Lei PAN, Chengrui LIU, Wangping LI

**Affiliations:** 710038 西安，空军军医大学唐都医院呼吸与危重症科; Department of Pulmonary and Critical Care Medicine, Tangdu Hospital, Air Force Medical University, Xi'an 710038, China

**Keywords:** 肺肿瘤, 循环肿瘤DNA, 分子残留病灶, 中枢神经系统转移, 颅内无复发生存, Lung neoplasms, Circulating tumor DNA, Molecular residual disease, Central nervous system metastasis, Intracranial recurrence-free survival

## Abstract

肺癌是全球发病率和死亡率最高的恶性肿瘤之一，其中非小细胞肺癌（non-small cell lung cancer, NSCLC）占绝大多数。患者术后中枢神经系统（central nervous system, CNS）转移预后极差，亟需更精准的分子标志物。基于循环肿瘤DNA（circulating tumor DNA, ctDNA）的分子残留病灶（molecular residual disease, MRD）检测虽能预测术后复发，但对CNS转移的预测价值存在争议。本文重点探讨MRD状态与NSCLC术后CNS复发风险的关联，梳理现有证据中MRD状态与总生存（overall survival, OS）的关系，分析外周血MRD对CNS转移产生“假阴性”的机制及临床对策。现有证据表明，MRD阳性与总体复发风险升高及OS缩短显著关联；部分研究提示CNS复发比例可能增加，但目前直接针对颅内无复发生存（intracranial recurrence-free survival, iRFS）的独立证据尚不充分。MRD阴性患者整体预后良好，但部分高危患者[表皮生长因子受体（epidermal growth factor receptor, EGFR）突变、III期、腺癌]仍可发生孤立性CNS转移，主要因血脑屏障阻碍ctDNA释放导致“假阴性”。奥希替尼辅助治疗可降低CNS复发风险，但停药后第1年内复发风险仍较高。MRD状态是NSCLC术后重要的预后分层工具，但外周血MRD阴性不能完全排除CNS复发风险，需结合影像学、高危因素及脑脊液检测综合评估。

## Abstract (en)

Lung cancer is one of the malignancies with the highest incidence and mortality rates worldwide, with non-small cell lung cancer (NSCLC) accounting for the vast majority. Patients with postoperative central nervous system (CNS) metastasis have a dismal prognosis, highlighting the urgent need for more precise molecular biomarkers. Although circulating tumor DNA (ctDNA)-based molecular residual disease (MRD) testing can predict postoperative recurrence, its utility in detecting CNS metastasis remains debated. This review examines the association between MRD status and CNS recurrence risk, synthesizes evidence linking MRD status to overall survival (OS), and explores mechanisms underlying false-negative results of peripheral blood MRD in CNS metastasis detection alongside potential clinical strategies. Current evidence indicates that MRD positivity correlates with increased overall recurrence risk and shortened OS; some studies suggest a potential increase in CNS recurrence, yet direct evidence specifically targeting intracranial recurrence-free survival (iRFS) remains insufficient. MRD-negative patients generally have favorable prognosis; however, a subset of high-risk patients - those with epidermal growth factor receptor (EGFR) mutations, stage III disease, or adenocarcinoma - may still develop isolated CNS metastases, primarily due to the blood-brain barrier limiting ctDNA release. Adjuvant Osimertinib reduces CNS recurrence risk, though recurrence remains high within the first year after treatment discontinuation. MRD status serves as an important prognostic stratification tool after NSCLC resection, but negative peripheral blood MRD does not completely exclude CNS recurrence risk. Comprehensive assessment integrating imaging, risk factors, and cerebrospinal fluid testing is therefore warranted.

非小细胞肺癌（non-small cell lung cancer, NSCLC）是发病率最高的恶性肿瘤之一，手术切除是早期核心治疗手段。一项基于纪念斯隆凯特琳癌症中心（Memorial Sloan Kettering Cancer Center, MSKCC）1294例早期NSCLC患者的研究^[1]^显示，术后5年内复发或第二原发肺癌的发生率约为27%。其中中枢神经系统（central nervous system, CNS）转移预后极差：脑转移患者中位总生存（overall survival, OS）仅约6.08个月^[2]^；未经治疗者仅数周至数月^[3]^；累及脑膜者仅约4.2个月^[4]^。

术后常规随访主要依赖影像学检查，但计算机断层扫描（computed tomography, CT）难以早期发现微小残留病灶，增强磁共振成像（magnetic resonance imaging, MRI）对脑膜转移灵敏度有限，且更密集的影像学随访未明确改善患者预后^[5]^，亟需更灵敏的监测手段。基于循环肿瘤DNA（circulating tumor DNA, ctDNA）的液体活检技术可检测术后外周血中的分子残留病灶（molecular residual disease, MRD）。研究^[6,7]^证实，术后ctDNA-MRD状态是NSCLC复发和生存的强效预后生物标志物。

然而，MRD检测在CNS转移预测中的价值存在争议。血脑屏障（blood-brain barrier, BBB）由紧密连接的毛细血管内皮细胞构成，严格限制大分子物质（包括ctDNA）的双向通过。CNS内转移灶释放的ctDNA难以穿越BBB进入外周血，导致“假阴性”。Zhang等^[8]^的前瞻性研究中，5例单纯脑部复发患者仅1例（20%）通过外周血MRD检出。

本文旨在重点探讨MRD状态与NSCLC术后CNS复发风险的关联，梳理现有证据中MRD状态与OS的关系，分析外周血MRD对CNS转移产生“假阴性”的机制，并提出临床应对策略。

## 1 MRD检测的技术基础与临床验证

### 1.1 ctDNA-MRD的检测方法

ctDNA是肿瘤细胞释放至体液的游离DNA片段，可通过液体活检检测。目前ctDNA-MRD检测主要分为两类策略：肿瘤知情（tumor-informed）与肿瘤未知（tumor-naive）^[9]^。

肿瘤知情策略通过对手术肿瘤组织测序，筛选患者特异性突变位点，在术后血浆中追踪，灵敏度高、背景噪音低。TRACERx研究^[10]^采用定制化多重聚合酶链式反应（polymerase chain reaction, PCR）面板，每例患者中位追踪200个突变，特异性>99%。超灵敏检测技术进一步提升了肿瘤知情策略的检测性能。以TRACERx项目采用的NeXT Personal平台为例，该技术通过追踪每例患者中位约1800个肿瘤特异性体细胞变异，结合分子共有序列降噪技术，可将ctDNA检测下限推至1-3 ppm，特异性达99.9%。在171例早期NSCLC的术前验证中，该平台将肺腺癌的术前ctDNA检出率从14%提升至81%，其中53%的病理I期患者可实现术前检出^[11]^。

肿瘤未知策略使用固定化panel检测常见肺癌驱动基因或甲基化标志，无需肿瘤组织信息，操作简便，报告周期短。Rosenlund等^[12]^采用商品化197基因panel对45例I-III期NSCLC患者（其中手术患者32例）进行治疗后MRD检测，在术后中位6.1个月的时间点，该策略预测复发的敏感性为50%，特异性为92.9%，在手术亚组中，治疗后早期（中位3.0个月）的ctDNA阳性与复发风险显著关联[比值比（odds ratio, OR）=11.8, P=0.0432]。这一结果表明，肿瘤未知策略在确保高特异性的前提下，对早期或低肿瘤负荷患者的灵敏度仍有待提升。

### 1.2 MRD的预后价值：检测性能与领先时间

术后ctDNA-MRD状态是NSCLC复发和生存的生物标志物之一，其价值体现在领先影像学复发时间、检测性能及风险量化3个方面。（1）领先时间：多项研究证实ctDNA-MRD可比影像学提前预测复发。Chaudhuri等^[13]^采用CAPP-Seq技术发现，ctDNA领先影像学进展中位时间为5.2个月，72%的患者更早检出复发。Gale等^[14]^的LUCID研究（n=88）报告领先时间212.5天，标志点ctDNA阳性患者复发风险比（hazard ratio, HR）高达14.8。Xia等^[15]^的综述汇总领先时间为3.0-6.7个月。（2）检测性能：Pellini等^[9]^总结，Landmark检测预测复发的敏感性为36%-100%，特异性为71%-100%；持续监测敏感性可提高至82%-100%，特异性为70%-100%。ADAURA MRD分析^[16]^显示，肿瘤知情检测敏感度为65%，特异度为95%，阳性预测值为91%，阴性预测值为78%，领先中位时间为4.7个月。TRACERx项目2025年发表于Cell的研究^[17]^采用超灵敏平台（检测下限1-3 ppm），对431例I-III期NSCLC患者进行动态监测，Landmark窗口（术后10-120天）内≥80 ppm阈值下预测复发敏感性为62%，下调后可提升至71%；Landmark时ctDNA<80 ppm的患者构成“中等风险”亚群，最佳分层阈值集中在60-90 ppm。Black等^[11]^在同一队列中发现，术前ctDNA<80 ppm的肺腺癌患者5年OS为61.4%，显著低于ctDNA阴性者的100%（HR=12.33, P=0.0029）。（3）风险量化：多项meta分析提供了合并效应量。Chen等^[18]^（28项研究，n=1686）显示术后ctDNA阳性与更差的无病生存期（disease-free survival, DFS）有关（HR=6.52），纵向持续阳性者HR达12.33。Lu等^[19]^（30项研究，n=3287）证实纵向监测敏感度（78%）优于Landmark（44%）。Zala等^[20]^（7项研究）按术后时间分层，术后3个月以上检测的HR（6.62）高于术后1周内（5.91）。Che等^[21]^（10项研究，n=1859）汇总MRD阳性患者的无进展生存期（progression-free survival, PFS）（HR=11.19）和OS（HR=6.34），亚组分析显示纵向监测HR（17.52）远高于Landmark（8.93）。此外，Peng等^[22]^的单中心前瞻性研究亦显示，术前ctDNA阳性患者的复发HR为3.812、死亡HR为5.004。

TRACERx 2025年研究^[17]^将术前与术后Landmark ctDNA状态联合分析，进一步细化风险分层：术前阴性+术后阴性组预后最佳，术前阳性+术后转阴组为中等风险（HR=2.88, P=0.035），术前阳性+术后仍阳性组预后最差（HR=0.18, P=3.57×10^-8^）。这表明术前ctDNA状态蕴含独立预后信息，将其纳入术后风险评估有助于精准识别需强化监测或治疗的患者。有meta分析^[21]^显示，纵向动态监测合并HR（17.52）显著高于Landmark（8.93）。LUNGCA^[23]^研究（n=233）显示，术后MRD阳性患者的3年复发阳性预测值（positive predictive value, PPV）为90.0%，阴性预测值（negative predictive value, NPV）为90.1%，无复发生存（recurrence-free survival, RFS）HR为18.7（95%CI: 10.4-33.4）；综合治疗（手术+辅助治疗）后MRD阳性患者的PPV达100%，NPV为90.3%，HR为30.5（95%CI: 16.1-58.0）。

### 1.3 MRD阴性定义的极低复发风险人群与停药后风险

持续MRD阴性可提示极低复发风险，但仍需警惕孤立性CNS复发的可能。Zhang等^[8]^的前瞻性研究（n=261）显示，持续MRD阴性的NPV达96.8%，仅3.2%的患者复发。MRD阴性患者无论是否接受辅助治疗，生存结局无显著差异，提示该亚组患者从辅助治疗中获益可能有限。

然而，停药后复发风险不容忽视。ADAURA MRD分析^[16]^显示，奥希替尼组87%的患者保持MRD阴性，但68%的复发或MRD事件发生在停药后，其中58%在停药后12个月内，提示停药后的持续监测仍然必要。

## 2 MRD状态与全身复发的关联

在探讨CNS“监测盲区”之前，需先明确MRD对全身（颅外）复发的预后价值。术后ctDNA-MRD状态是NSCLC全身复发的最强预测因子之一^[9]^，对复发风险、时间及辅助治疗获益具有显著分层作用。

### 2.1 MRD阳性：复发风险高、复发时间短

Verzè等^[24]^的综述（13项研究）显示，术后MRD阳性患者复发风险显著升高，RFS和OS缩短；MRD可比影像学平均提前5.5个月预测复发。Pellini等^[9]^报道，Landmark检测敏感性为36%-100%，特异性为71%-100%；持续监测敏感性可提升至82%-100%。

Moding等^[25]^在局部晚期NSCLC中发现，放化疗后MRD阳性者100%在12个月内复发，领先影像学4.1个月。TRACERx研究^[10]^对197例I-III期患者进行纵向监测，Landmark分析显示25%的患者MRD阳性，其中49%最终复发；MRD领先影像学复发中位228天（约7.6个月）。

### 2.2 MRD阴性：整体良好，但需警惕例外

Zhang等^[8]^的研究显示，MRD阴性患者整体预后良好（NPV达96.8%），但MRD转阳风险高峰在术后18个月，此后逐步下降。MRD阴性患者无论是否接受辅助治疗，生存无显著差异。Frisone等^[26]^指出，ctDNA阴性患者可能避免不必要的辅助化疗，但需前瞻性验证。

然而，MRD阴性并非绝对安全。ADAURA MRD分析^[16]^显示，奥希替尼组87%保持MRD阴性，但仍有少数患者复发，部分为CNS孤立性转移。Xia等^[27]^（LUNGCA-1）显示，MRD阳性患者可从辅助治疗显著获益（P=0.002），而MRD阴性患者PFS无差异（P=0.283）。Che等^[21]^的meta分析证实，MRD阳性患者辅助治疗获益显著（HR=0.27），而MRD阴性未获益（HR=1.51）。上述结果提示，MRD状态可指导辅助治疗决策，但MRD阴性不等于“零风险”，尤其是对CNS转移的监测不可放松。

### 2.3 全身复发模式的差异：MRD阳性与阴性的不同特征

TRACERx研究^[10]^显示MRD阳性患者复发多为远处转移（骨、肝、对侧肺）；MRD阴性患者复发相对局限，部分为局部/区域淋巴结或CNS。ADAURA MRD分析^[16]^同样显示，MRD阳性组远处转移比例更高，MRD阴性组局部或仅CNS复发比例更高。

### 2.4 小结：全身复发视角下的MRD价值

MRD状态对NSCLC术后全身复发具有强大预后价值：MRD阳性预示高复发风险，患者可能从辅助治疗中获益；MRD阴性整体良好，但不能完全排除局部区域和CNS转移。这一局限性将在第4节重点讨论。

### 2.5 证据异质性分析

上述研究虽一致表明ctDNA-MRD阳性与更高的复发风险有关，但具体效应量在不同研究中存在显著差异（PFS合并HR=11.19，I^2^=77.3%^[21]^），异质性主要源于以下因素：（1）检测策略与监测模式：肿瘤知情策略因追踪患者特异性突变，灵敏度普遍优于肿瘤未知固定panel^[9]^；纵向动态监测合并HR（17.52）显著高于Landmark（8.93）^[21]^。检测时间点同样影响效能：术后3天内可能混入手术创伤释放的预存ctDNA，术后1个月检测可更准确反映MRD^[28]^。（2）辅助治疗背景：辅助治疗[尤其是酪氨酸激酶抑制剂（tyrosine kinase inhibitors, TKIs）]可清除部分MRD阳性信号，使“治疗后MRD阴性”患者的预后被系统性优化^[23]^，不同研究中辅助治疗比例差异直接影响MRD的预后效应量。（3）组织学异质性：TRACERx系列研究^[11]^显示，ctDNA的预后价值在非肺腺癌（即鳞癌等非腺癌组织学类型）中显著弱于腺癌。（4）CNS转移的报告差异：多数研究仅报告总体DFS/PFS，未将CNS复发作为独立终点分析^[29]^；部分研究虽提及CNS复发，但未区分脑实质转移与脑膜转移，后者脑脊液（cerebrospinal fluid, CSF）ctDNA检出率远高于外周血^[15]^。这一报告标准的不统一，加之现有研究多将CNS复发归入总DFS/PFS事件而未进行独立分析^[29]^，这是“MRD对CNS转移预测价值”结论不一的核心原因。

## 3 外周血MRD对CNS转移的监测盲区

外周血ctDNA-MRD阴性对全身复发具有较高的NPV（达96.8%），持续MRD阴性可提示极低复发风险^[8]^。然而，临床证据表明，MRD阴性并不能完全排除CNS转移风险，这一“陷阱”源于BBB对ctDNA释放的物理阻碍。

### 3.1 临床证据

外周血MRD对CNS转移的检测灵敏度严重不足。Zhang等^[8]^报道，5例单纯脑部复发患者仅1例（20%）通过外周血MRD检出。Tang等^[7]^同样指出，NSCLC脑转移患者外周血MRD阳性检出率仅约20%。ADAURA MRD分析^[16]^虽未专门分析CNS转移，但其停药后复发高峰数据具有提示意义：奥希替尼组68%的DFS或MRD事件发生在停药后，其中58%在停药后12个月内。

TRACERx项目2025年的超灵敏ctDNA监测数据为上述“CNS监测盲区”提供了直接的量化证据。全部复发患者中，胸腔内或脑部孤立复发者复发时的外周血ctDNA中位水平仅为颅外复发者的1.5%（293 vs 19,496 ppm, P=7.1×10^-6^）；复发时ctDNA仍为阴性的患者中，100%（11/11）为胸腔内或脑部孤立复发，而ctDNA阳性复发者中仅56%（37/67）为此类复发^[17]^。该研究还发现，56.5%的Landmark MRD阴性复发患者存在微乳头亚型（校正HR=5.21，P=0.005）。

LUNGCA研究^[23]^全程结果进一步验证了上述规律。34例复发后采集血浆样本的患者中，18例（52.9%）复发后ctDNA呈阴性；复发后ctDNA阴性者中位OS为40.9个月，显著优于阳性者的19.4个月（HR=0.4, P=0.029）。复发后ctDNA阴性患者中83.3%（15/18）为脑转移或局部复发，而ctDNA阳性患者中75.0%（12/16）为骨转移或多器官复发。

此外，TRACERx 2025年研究^[17]^观察到，复发时外周血ctDNA仍为阴性的肺腺癌患者中，携带表皮生长因子受体（epidermal growth factor receptor, EGFR）/间质-上皮细胞转化因子（mesenchymal-epithelial transition factor, MET）/间变性淋巴瘤激酶（anaplastic lymphoma kinase, ALK）等驱动基因阳性的比例显著增高（40.0% vs 8.8%, P=0.037），提示驱动基因阳性NSCLC患者术后即使外周血MRD持续阴性，仍应高度警惕CNS孤立复发风险。个案层面：Wang等^[30]^报道1例MET ex14跳跃突变局部晚期NSCLC患者，新辅助克唑替尼治疗后达病理完全缓解，术后ctDNA持续阴性，但停药后2个月内出现肺转移。Takahashi等^[31]^报道1例停用奥希替尼后出现脑膜转移的患者，头颅磁共振成像（magnetic resonance imaging, MRI）和CSF细胞学均呈阴性，最终通过CSF EGFR突变检测确诊。

### 3.2 外周血MRD对CNS转移不敏感的机制

BBB由脑毛细血管内皮细胞、基膜和周细胞构成，内皮细胞间以紧密连接相连，形成物理屏障，阻碍CNS来源ctDNA向外周血的释放。Mo等^[32]^系统阐述了BBB的分子结构，内皮细胞间以claudin-5、occludin、ZO-1等紧密连接蛋白形成高电阻屏障，P-gp和BCRP等外排泵进一步限制大分子入脑。

ctDNA在血液中的半衰期仅约2 h，其浓度与肿瘤负荷、增殖活性及脱落效率相关^[7]^。CNS转移灶（尤其是脑膜转移）的肿瘤负荷通常远小于全身性多发转移，加之BBB阻碍，释放至外周血的ctDNA浓度极低，常低于现有检测技术的极限[如0.02%突变丰度（variant allele frequency, VAF）]。动态监测显示，MRD阳性患者的ctDNA半衰期显著长于MRD阴性患者（103.2 vs 29.7 min, P=0.001）^[28]^。

此外，CNS转移灶可能起源于原发肿瘤中的特定亚克隆，其ctDNA脱落能力存在差异。约54.5%的患者在手术切除的肺组织残血中可检出MRD，而同期外周血检测为阴性^[7]^，表明部分肿瘤属于“非脱落”表型。技术灵敏度限制同样不可忽视：即使是最先进的肿瘤知情检测，仍存在检测下限，极低丰度的ctDNA可能被漏检。

### 3.3 CSF ctDNA检测

CSF ctDNA对CNS转移（尤其是脑膜转移）的灵敏度远高于外周血，可达80%-100%^[15]^。李羽斌等^[33]^发现：CSF中驱动基因检出率（100% vs 52%）和突变丰度（22.79% vs 0.57%）均显著优于外周血（P<0.001），且可检测到独特的拷贝数变异，与软脑膜转移和耐药相关。新近发布的《驱动基因阳性非小细胞肺癌脑膜转移临床诊疗中国专家共识（2026版）》^[34]^已将CSF液体活检提升至关键诊断地位，CSF ctDNA检测有助于明确NSCLC脑膜转移患者的驱动基因突变类型及疗效评估（A级推荐）；CSF ctDNA检测可作为细胞学阴性但高度怀疑脑膜转移患者的辅助诊断手段（B级推荐）。

尽管CSF ctDNA灵敏度显著优于外周血，但其临床应用仍面临多重局限：（1）腰椎穿刺为侵入性操作，难以作为常规筛查手段；（2）标本量有限，不同检测平台的阈值及质控标准尚未统一；（3）证据多来自回顾性研究或小样本研究，对无症状脑实质微转移的价值仍需验证；（4）CSF ctDNA对脑实质转移的灵敏度尚不明确。因此，CSF ctDNA更适合作为高度怀疑CNS转移（尤其软脑膜转移）患者的补充检测手段，而非替代外周血MRD或头颅增强MRI^[34]^。

### 3.4 临床对策

（1）影像学监测：对CNS转移高危患者（III期、腺癌、EGFR/ALK突变等），即使MRD持续阴性，可考虑每6-12个月行头颅增强MRI（停药后第1年内加密），但该建议尚需前瞻性验证。（2）症状导向评估：出现不明原因头痛、恶心、神经系统症状时，即使MRD阴性和头颅MRI阴性，在临床可行的情况下可考虑腰椎穿刺并送检CSF ctDNA。

## 4 临床启示与对策

### 4.1 风险分层：不能因MRD阴性而放松CNS监测

以下高危因素提示CNS转移风险增加，即使MRD阴性也应加强CNS监测：（1）病理分期：III期（尤其是N2）；（2）组织学类型：腺癌；（3）驱动基因状态：EGFR突变（ADAURA研究^[35]^安慰剂组CNS复发率为10%）、ALK/c-ROS肉瘤致癌因子-受体酪氨酸激酶（ROS proto-oncogene 1, receptor tyrosine kinase, ROS1）融合；（4）淋巴结转移：多站、N2受累；（5）病理亚型：微乳头成分、脉管癌栓。

Zhao等^[36]^的综述显示，EGFR突变患者3年累积脑转移发生率为29.4%-60.3%，显著高于野生型。Ohhara等^[37]^的多中心研究（HOT 1701, n=566）表明，驱动基因阳性（EGFR/ALK）同步脑转移患者中位OS达23.5个月，阴性者仅7.6个月（P<0.001）；独立预后因素包括年龄≥65岁、美国东部肿瘤协作组（Eastern Cooperative Oncology Group, ECOG）卡氏体能状态（Karnofsky performance status, KPS）评分2-4分、T3-T4分期、无驱动基因突变、存在颅外转移、脑转移数量≥4个。

### 4.2 监测策略升级

（1）影像学监测：高危患者即使MRD阴性，可考虑每6-12个月行头颅增强MRI，停药后第1年内可适当加密，但此建议基于现有证据的专家推论，尚缺乏前瞻性研究验证其对预后的改善作用。（2）ctDNA监测时机：专家共识建议术后首次检测在根治性切除后1周至1个月内，之后每3-6个月复查^[7]^。Zala等^[20]^的meta分析显示，术后3个月以上检测的HR（6.62）高于术后1周内（5.91），提示远期监测同样重要。Peng等^[22]^报道术后ctDNA领先影像学复发中位12.6个月，89.5%的术后阳性患者在术后2周内检出。（3）症状导向评估与CSF检测：出现不明原因头痛、恶心、神经系统症状时，即使MRD阴性、头颅MRI阴性，也应考虑腰穿+CSF ctDNA检测。CSF常规、细胞学、ctDNA是确诊关键。

上述监测策略建议主要基于回顾性研究及专家经验，目前尚无前瞻性随机对照试验验证以MRD状态指导的强化监测能否改善NSCLC术后CNS转移患者生存预后。临床应用中应结合患者个体情况、医疗资源及经济因素综合决策，有条件者可积极参与相关临床试验^[29]^。

### 4.3 治疗决策考量

（1）停药决策：MRD阴性不应作为停用辅助靶向治疗的唯一依据。ADAURA研究^[35]^确立了3年奥希替尼为标准，ICOMPARE研究^[38]^显示2年优于1年。停药需综合分期、高危因素及治疗时长，若需降低CNS复发风险，应优先考虑高CNS穿透性TKIs。（2）高CNS穿透性TKIs：奥希替尼（ADAURA^[35]^）、阿来替尼（ALINA）等可显著降低CNS复发风险。在ALK阳性NSCLC中，III期ALINA研究^[39]^显示，辅助阿来替尼24个月可使II-IIIA期患者2年PFS率从63.0%提升至93.8%（HR=0.24），CNS复发或死亡风险降低78%（HR=0.22）。Shalata等^[40]^的研究显示，奥希替尼颅内客观缓解率（objective response rate, ORR）达76%，中位颅内PFS为8-15个月。Gil等^[41]^指出，新一代ALK抑制剂（阿来替尼、布格替尼、劳拉替尼）CSF/血浆比可达0.75，显著降低CNS进展风险（HR=0.16）。2026版中国专家共识^[34]^确立了EGFR突变阳性合并脑膜转移患者一线优选奥希替尼、伏美替尼等高CNS渗透性TKIs（A级推荐），ALK阳性患者优先推荐洛拉替尼或阿来替尼。

LUNGCA研究^[23]^显示，166例可靶向突变患者中，辅助治疗前ctDNA阳性者接受TKIs治疗后仅25%复发，化疗或不治疗者均复发（HR=0.01, P=0.005）；67例无靶向突变患者中，辅助化疗未能改善RFS（HR=0.2, P=0.093）。该结果表明，辅助治疗前ctDNA状态与肿瘤基因型联合分析可预测不同辅助治疗方案疗效，为MRD指导个体化辅助治疗提供了依据。

## 5 未来展望

有共识^[34]^首次将CSF分子活检列为驱动基因阳性NSCLC脑膜转移的核心诊断依据之一，为构建“外周血MRD监测全身负荷+CSF ctDNA监测颅内微环境”的双轨动态监测体系提供了循证支持。

### 5.1 CSF ctDNA：突破CNS监测盲区的关键方向

CSF ctDNA对CNS转移（尤其是脑膜转移）的诊断敏感性远高于外周血。Liang等^[42]^的前瞻性研究纳入25例NSCLC软脑膜转移患者，CSF循环游离DNA（cell-free DNA, cfDNA）下一代测序检出驱动突变率达100%（包括6例细胞学阴性患者），72%的患者据此调整了治疗方案。有研究^[43]^指出，CSF ctDNA对软脑膜转移的诊断敏感性可达80%-100%。一项纳入26项研究的meta分析^[44]^显示，CSF ctDNA汇总检测率（86%）显著优于传统细胞学（60%）。然而，CSF ctDNA的临床应用还需解决标准化检测流程、前瞻性验证无症状人群获益及卫生经济学评价等问题，在现阶段应严格把握适应人群，避免过度使用。

### 5.2 “外周血+CSF”双轨监测体系

对CNS转移高风险患者（如EGFR突变III期腺癌），可建立分层监测：（1）常规随访：外周血MRD+头颅增强MRI；（2）可疑CNS症状时：升级为CSF检测。

### 5.3 MRD指导的适应性治疗策略

（1）降级策略：持续MRD阴性（尤其术后18个月后）的低危患者，可探讨缩短辅助治疗或减少监测频率。正在进行的CTONG 2201研究（NCT05457049）是首个前瞻性评估基于连续2次术后MRD阴性省略辅助治疗策略的临床试验，计划入组180例IB-IIIA期患者，以2年DFS率为主要终点^[45]^。（2）升级策略：MRD阳性或高危患者，延长辅助治疗、加强CNS监测、考虑联合治疗。APPROACH/CTONG2101研究（NCT04841811）^[46]^是一项针对EGFR突变阳性不可切除III期NSCLC的随机III期试验，探索阿美替尼诱导治疗后基于动态MRD监测指导后续治疗的策略，主要终点为ORR和无事件生存（event free survival, EFS）率。Abbosh等^[29]^提出ctDNA指导的适应性治疗框架：MRD阳性应治疗升阶，MRD阴性可考虑降阶，但需警惕局部或CNS复发的“盲区”。

## 6 总结

MRD状态是NSCLC术后重要的预后分层工具。现有证据明确支持MRD阳性与全身复发风险升高及OS缩短显著关联；在CNS复发预测方面，目前证据主要指向外周血MRD对CNS转移存在监测盲区（敏感性不足），直接针对颅内无复发生存（intracranial recurrence-free survival, iRFS）的独立证据尚不充分。因此，MRD状态的核心临床应用应定位于复发风险分层，而非CNS转移的特异性预测。

MRD阴性患者整体预后良好，持续MRD阴性可提示极低复发风险（“潜在治愈”倾向），但单次MRD阴性不能排除后续CNS转移可能。由于BBB的阻碍，外周血MRD阴性不能完全排除CNS转移风险，尤其是III期、EGFR突变、腺癌等高危患者。临床决策应综合MRD动态变化、影像学、病理高危因素及驱动基因类型。

基于MRD动态变化的复发风险分层管理可从以下维度推进：（1）风险识别：持续MRD阴性提示极低复发（NPV 96.8%），需警惕术后18个月转阳高峰；MRD阳性则高危。（2）监测强化：MRD阳性或高危因素患者可考虑每6-12个月行头颅增强MRI。（3）治疗决策：MRD阴性非停药唯一依据，需综合分期及高危因素；MRD阳性优先考虑高CNS穿透性TKIs。（4）动态随访：建议术后3-6个月复查ctDNA-MRD，持续阴性者间隔可延长，出现神经系统症状时升级为CSF检测。

对CNS高风险患者，即使MRD阴性，也应规律行头颅增强MRI，出现神经系统症状时积极行CSF检测。未来，“外周血+CSF”双轨监测体系及MRD指导的适应性治疗策略有望进一步优化NSCLC术后管理。
